# Supplementary material for: Assessment of the Anti-Thrombogenic Activity of Polyurethane Starch Composites
Source: J Funct Biomater. 2022 Oct 12;13(4):184. doi: 10.3390/jfb13040184 (PMC9589968; doi:10.3390/jfb13040184)
Supplement: Supplementary file 1 [file jfb-13-00184-s001.zip › jfb-1885214-supplementary.pdf]

Supplementary material

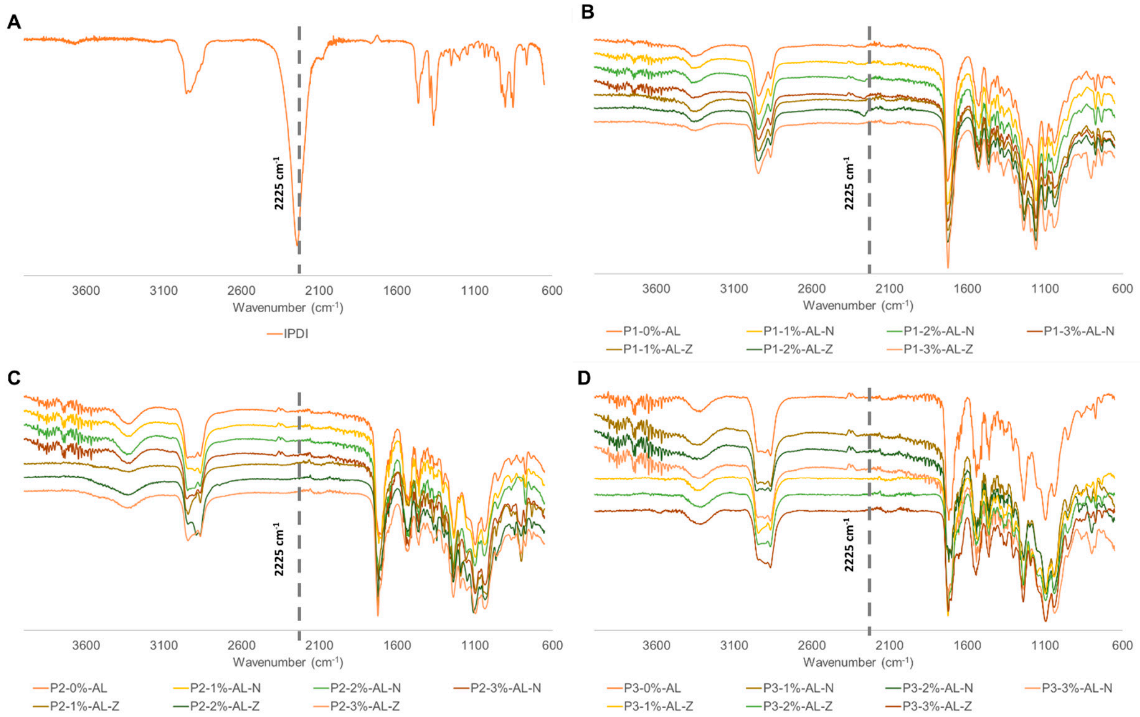

Figure S1. FTIR spectra of IPDI and polyurethane composites. Spectra of (A) IPDI, (B) P1, (C) P2, and (D) P3 of polyurethane composites. This figure was adapted from the paper Influence of Starch on the Structure-Properties Relationship in Polyethylene Glycol/Polycaprolactone Diol Polyurethanes. [26] with the authorization of the authors.

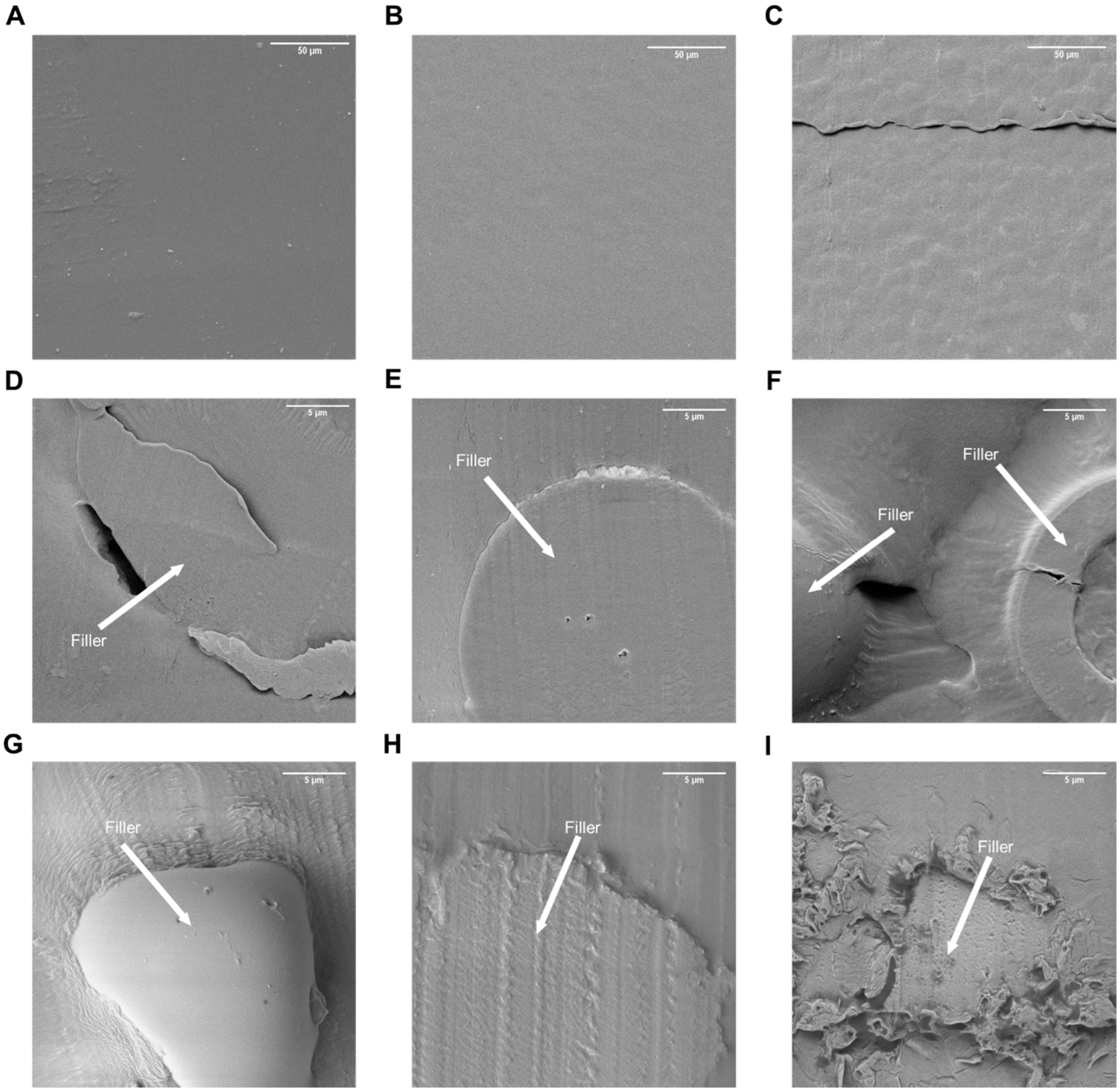

Figure S2. SEM images of polyurethane matrices and composites. Representative images of (A) P1-0%-AL, (B) P2-0%-AL, (C) P3-0%-AL, (D) P1-3%-AL-N, (E) P2-3%-AL-N, (F) P3-3%-AL-N, (G) P1-3%-AL-Z, (H) P2-3%-AL-Z, and (I) P3-3%-AL-Z. This figure was adapted from the paper Influence of Starch on the Structure-Properties Relationship in Polyethylene Glycol/Polycaprolactone Diol Polyurethanes. [26] with the authorization of the authors.

Table S1. Energy dispersive X-ray spectroscopy analysis. Representative Element Weight Composition of polyurethane composites. This table was adapted from the paper Influence of Starch on the Structure-Properties Relationship in Polyethylene Glycol/Polycaprolactone Diol Polyurethanes. [26] with the authorization of the authors.

PU	Starch	Concentration	Zone	Element Wt%		
				C	O	S

	AL-N			71.05	28.95	0
	AL-Z			66.01	28.52	4.02
P1		AL-0%		87.95	12.05	0
	AL-N	3%	Matrix	85.61	14.39	0
			Filler	80.37	19.63	0
	AL-Z	3%	Matrix	85.73	12.56	0
			Filler	79.11	19.22	1.20

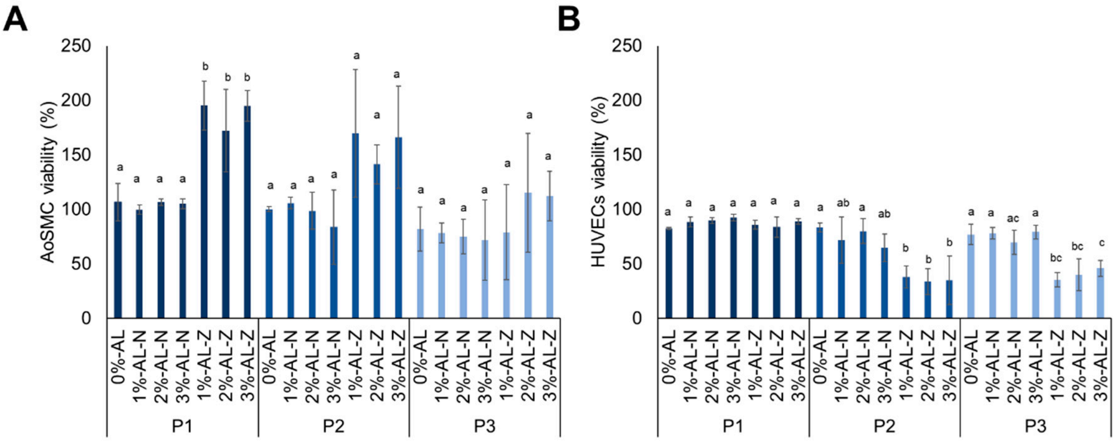

Figure S3. Cell viability assay of composites. (A) Cell viability of AoSMC and (B) HUVECs.
